# Vaginal Evisceration of Small Bowel With Extraperitoneal Ileal Resection of the Herniated Loops: A Case Report

**DOI:** 10.3389/fsurg.2022.878760

**Published:** 2022-04-26

**Authors:** Giulia Missori, Nicolò Luigi Arrigo Marchesini, Donatella Mosca, Andrea Aurelio Ricciardolo, Francesco Serra, Roberta Gelmini

**Affiliations:** ^1^Department of Surgery, Azienda ospedaliero-Universitaria Policlinico di Modena, Modena, Italy; ^2^Department of Emergency Medicine, Azienda ospedaliero-Universitaria Policlinico di Modena, Modena, Italy

**Keywords:** emergency surgery, hernia surgery, transvaginal evisceration, pelvic organ prolapse (POP), pelvic floor disorders

## Abstract

**Introduction:**

Vaginal evisceration is an extremely rare surgical emergency that can be described as the extrusion of abdominal viscera through a defect or a rupture of the vaginal wall. We reported the case of an acute abdomen due to small bowel evisceration secondary to vaginal vault dehiscence that required combined vaginal-abdominal approach

**Case:**

We discuss the case of a 72-year-old female who presented to the emergency department for a large prolapse with visible extrusion of the small bowel per vagina. The eviscerated bowel was resected by external vaginal approach due to excessive swelling of the loops which made it impossible to reduce them through the vagina defect. A midline laparotomy was undertaken for further assessment, and the vault defect was closed by transabdominal repair

**Conclusion:**

From its first description in 1864, just a few cases of vaginal evisceration had been described in the medical literature; the most common organ to eviscerate is the distal ileum, although cases of omentum, colon, fallopian tube, and appendix evisceration have also been reported. We described a rare case of transvaginal evisceration of the small bowel in our emergency department; it is a rare surgical emergency that must be managed to prevent serious consequences, such as bowel ischemia and necrosis, sepsis, and death. We suggest that a multidisciplinary approach to prompt examination and management by gynecologists and general surgeons is recommended to reduce the risk of morbidity and mortality. With this paper the authors would like to share the surgical manage of this rare emergency with other surgeons all around the world.

## Introduction

Vaginal evisceration is a surgical emergency that can be described as the extrusion of abdominal viscera through a defect or a rupture of the vaginal wall. The distal ileum is usually the eviscerated organ (1). It is a rare condition but potentially a life-threatening emergency requiring emergency surgery to prevent morbidity as bowel perforation, necrosis, and secondary sepsis (2). Since the first report by Hyernaux in 1864 (3), just a few cases have been described in medical literature, and the exact incidence is still challenging to evaluate. A small number of isolated cases are presented with exposed bowel loops protruding through the vagina. More often, transvaginal eviscerations are more subtle on presentation as found by Croak et al. that just a third of patients presented with visibly incarcerated bowel (4). Significant risk factors are a clinical history of hysterectomy in postmenopausal woman, vaginal vault dehiscence and recurrent episodes of prolapse (cystocele and rectocele) (5), which can be worsened by some conditions such as suddenly raised intra-abdominal pressure and vaginal trauma (6). The management of vaginal evisceration involves different approaches of emergency surgery (abdominal, vaginal, or combined) to replace the bowel into the peritoneal cavity. Rarely there is the need for resection (7).

The aim of this paper is to report the case of a patient presenting with acute abdomen due to small bowel evisceration secondary to vaginal vault dehiscence that required combined vaginal-abdominal approach with bowel resection performed through the vagina.

## Case Presentation

A 72-year-old patient had access to our emergency room complaining of a painful mass protruding through the vagina and concomitant generalized abdominal pain after evacuation happened on the morning of the same day. The patient reported a surgical history of transvaginal hysterectomy 12-years before due to a benign condition, with no surgical difficulties or postoperative complications reported. The patient did not assume any drugs.

On admission, vital signs were relatively normal (BP 150/80 mmHg, HR 100 beats per minute, 99% saturation in ambient, temperature 36°C), and the blood results showed: Ph 7.5, lactate 1.0 mmol/l, white cell count 9 migl./mmc, C-reactive protein 0.5 mg/dl, hemoglobin 13.9 g/dl. Clinical findings included dry oral mucosa, distended abdomen with absent bowel sounds, hypogastric tenderness whit no signs of peritoneal irritation, and a general condition of intense pain.

**Figure 1:**
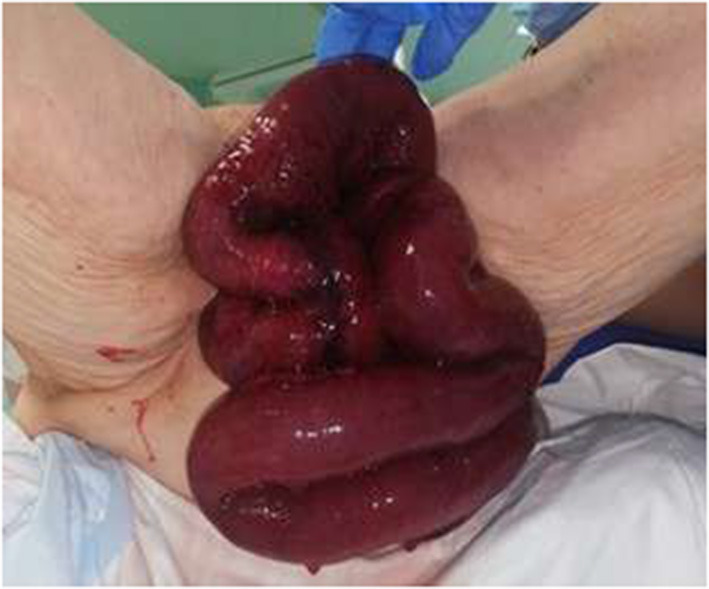
Necrotic ileal loops eviscerated from the vagina.

The patient was referred to the general surgeon for examination, revealing multiple edematous and necrotic loops of small bowel protruding through the vaginal vault, with part of the mesentery (Figure 1). The digital rectal exam shows that there was no involvement of the rectovaginal sectum. The exposed mass was immediately covered with sterile gauze moistened with warm saline solution. A nasogastric tube was positioned resulting in drainage of 400 cc of enteric fluid and a bladder catheter was placed.

The patient was in great pain, so a first support therapy whit analgesics (diazepam 10 mg ½ fl + paracetamol 1gr 1fl + fentanyl 0.1 mg 1 fl) and antiemetics (ondansetron 4 mg 1 fl) were started immediately. A pre-operative therapy whit intravenous fluid resuscitation and broad-spectrum antibiotics (piperacillin-tazobactam, 9gr) was started, and the patient was carried to the operating room for emergency surgery.

**Figure 2:**
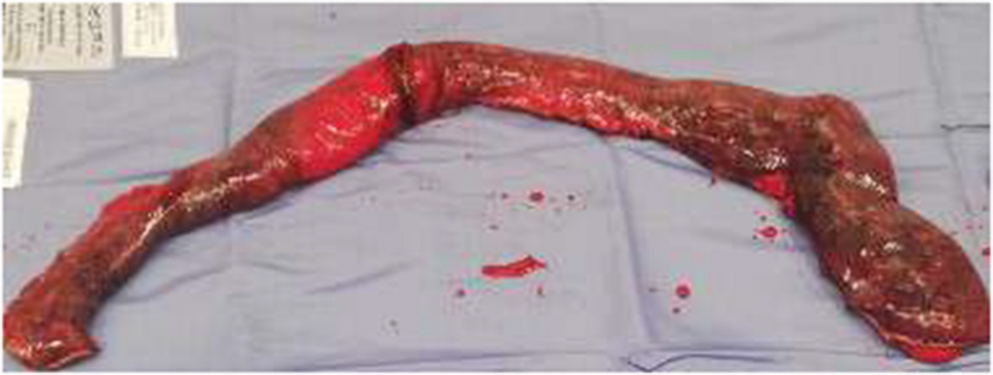
Macroscopic aspect of the ileum resected.

**Figure 3:**
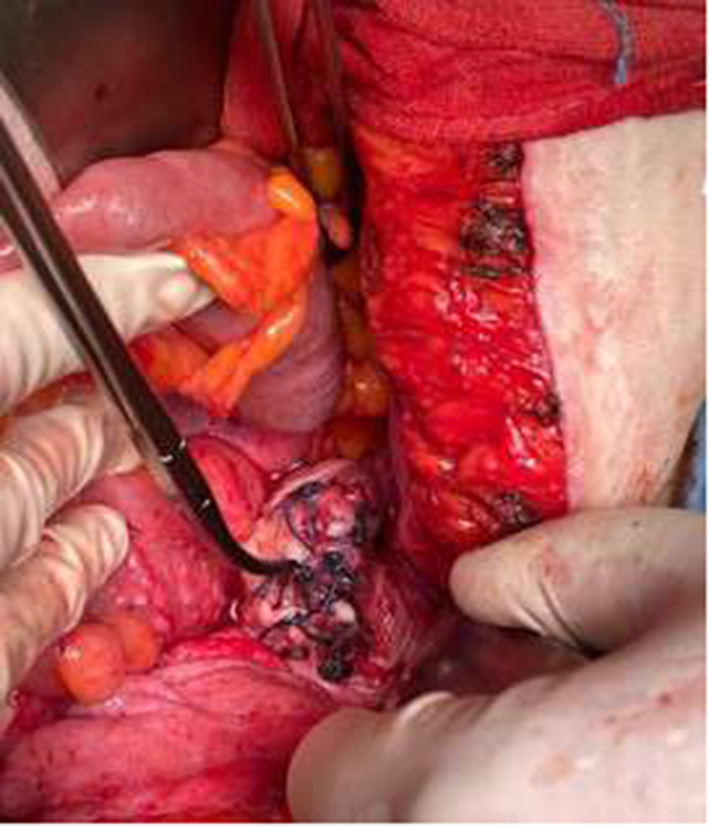
Interrupted stiches on the vagina.

A midline laparotomy was performed, and the small bowel was gently manipulated from the Treitz ligament until the herniated loops were identified. The prolapsed mass, composed of about 50 cm of ileal loops and their mesentery, appeared edematous and frankly necrotic, with no pulse palpable or apparent peristalsis. A manual transvaginal reduction was revealed impossible because of the tightness of the vaginal vault defect and the voluminous mass herniated, despite an enterotomy being performed to empty the bowel. After a first accurate evaluation, it was clear that an endoabdominal resection of the necrotic loops was not possible because a secure vascular control could not be obtained, so it was mandatory to have an external approach. The herniated necrotic loops were carefully isolated and resected, and after this, it could be possible to relocate back the rest of the vital ileum in the peritoneal cavity. Once in the abdomen, the ileum left appeared vital and vascularized well. During the inspection of the ileum, we noticed a Meckel diverticulum close to the previous resection (Figure 2). The Meckel was resected, and a hand-sewn ileo-ileal latero-lateral anastomosis was made. The vaginal vault defect was closed by interrupted suture by the colleague gynecologist (Figure 3); the plan by plan reconstruction of pelvic floor was not possible in such emergency condition. Also, if we were pretty sure about the integrity of the bladder, we performed the same methylene blue test from the bladder catheter, which was negative.

The patient spent 1 day in the intensive care unit for vital signs control. No surgical or clinical complication, like surgical site infection, was observed during the hospital stay and the patient was discharged after seven day from surgery. The gynecologist has scheduled a follow up a month away to place a pessary to reduce the risk of future vaginal prolapse.

## Discussion

From its first description in 1864, just a few cases of vaginal evisceration had been described in medical literature. It remains a rare condition that occurs mainly after vaginal surgery, with a peak rate after hysterectomy (8). As described by Rana et al., considering all the cases reported in the literature, 70% of all patients were postmenopausal women; of those, 73% had previous vaginal surgery, and 63% had enterocele. This increased incidence in postmenopausal women has been associated with general atrophy of the vaginal wall caused by different conditions. Enterocele, pelvic flow weakness with chronic prolapse, chronic steroid therapy, poorly controlled diabetes, hypoestrogenism, prior radiation therapy, and chronic renal insufficiency (9–11) are related to the atrophy of the vaginal wall. In this fragile condition, the triggering factor appears to be the increase of endoabdominal pressure mainly caused by obesity, ascites, constipation, or chronic respiratory pathology (asthma, BPCO). In premenopausal women, transvaginal evisceration is frequently associated with recent vaginal trauma. Particularly the early resumption of sexual intercourse is considered the most common precipitating event (12). Regarding surgical technique, laparoscopic approach is related to a higher incidence of vaginal cuff dehiscence (13).

The most commonly eviscerated organ is the distal ileum, although cases of omentum, colon, fallopian tube, and appendix evisceration have also been reported (8). Most vaginal vault defects are minor, making it easier to repair and prevent the prolapse of a too-long ileum segment. Morevoer, if the evisceration also included a portion of mesentery, this can rapidly lead to intestinal strangulation, as it happened in our case.

The reported median times from hysterectomy to evisceration are 3 months following totally laparoscopic, 6.5 months following abdominal, and 34 months following vaginal hysterectomy (14). In the case that we report, the evisceration happened after 12 years after surgery and just few cases describe an evisceration as long as 20 years or more after last pelvic surgery (15).

The patients commonly present with pelvic or lower abdominal discomfort, vaginal pain, bleeding, and feeling of fullness. Only sometimes they show obvious protruding viscera from the vagina (16, 17), as in our case report. As described by Ramirez et al., almost all presentations are subtle, with no specific symptoms and no clear evidence that can justify the suspect of an evisceration (9).

Once the diagnosis is made, the first management consists of stabilization, intravenous therapy of fluid resuscitation, and broad-spectrum antibiotics. The exposed viscera have to be covered with sterile gauze or towels moist by warm saline solution until transfer to the operating room as soon as possible for definitive repair. The primary aim of the surgical procedure is to examine the herniated loops to understand their vitality and resect the non-viable bowel. The abdominal approach via a midline laparotomy is the most used. It provides the best exposure for inspecting the abdominal viscera and irrigation, drainage, and resection of non-viable bowel. By the way an alternative vaginal and/or laparoscopic approach can be chosen. The vaginal approach may be appropriate if there is no sign of peritonitis, ischemic injury, or strangulation. The bowel can be reduced in the abdomen through the vagina; this technique, however, limits the bowel's examination in its total length. A combined laparoscopic and vaginal approach has the defect to evaluate the peritoneal cavity before the vaginal vault is recovered.

In our case we choose first a midline laparotomy approach, as it also recommended by literature. Nevertheless, it was no possible to resect the ileum with an abdominal approach because the concurrent herniation of the mesentery did not allow to have proper control of the vascular axis and at the same time the vaginal wall defect was very small, making it impossible to reduce the intestinal loops. This is the reason why we attempt the ileal resection directly from the vagina, with subsequent transabdominal examination of the resected ileum and closure of the vaginal defect.

We fully support the transabdominal approach for treating vaginal evisceration as this allows a better overview of the clinical picture. However, if it is not possible to proceed safely with this technique, different approches must be taken into account.

## Conclusion

We described a rare case of transvaginal evisceration of the small bowel in our emergency department; it is a rare surgical emergency that must be managed to prevent serious consequences, such as bowel ischemia and necrosis, sepsis, and death.

It is difficult to identify a patient whit increased risk. Still, it is a condition that should always be suspected in women with a clinical history of previous abdominal surgery, with greater attention to those that have undergone hysterectomy, despite the time elapsed.

The most cases reported in the literature present a clinical situation where the herniated loops are vital and easily reducible with no resection. In the presence of necrotic tissue, a bowel resection is mandatory, and endoabdominal surgery should be preferred. However, if this technique is not considered safe enough, external resection is the only option left.

## Data Availability Statement

The original contributions presented in the study are included in the article/supplementary material, further inquiries can be directed to the corresponding author/s.

## Author Contributions

GM and RG: paper compilation and initial surgical management. GM and NM: paper compilation and research. AR: reference compilation and edits. DM and FS: overseeing of project and primary surgeon. All authors contributed to the article and approved the submitted version.

## Conflict of Interest

The authors declare that the research was conducted in the absence of any commercial or financial relationships that could be construed as a potential conflict of interest.

## Publisher's Note

All claims expressed in this article are solely those of the authors and do not necessarily represent those of their affiliated organizations, or those of the publisher, the editors and the reviewers. Any product that may be evaluated in this article, or claim that may be made by its manufacturer, is not guaranteed or endorsed by the publisher.
